# Association of the apolipoproteins with retinal arteriosclerosis in a health examination population

**DOI:** 10.3389/fcvm.2026.1852420

**Published:** 2026-06-17

**Authors:** Xinghe Sun, Lijuan Guo, Hui Lv, Xian Zhang, Chaoqun Wu, Xiaohui Liu

**Affiliations:** 1 Department of Cardiology, Peking University International Hospital, Beijing, China; 2 Healthcare Management Center, Peking University International Hospital, Beijing, China; 3 National Clinical Research Center of Cardiovascular Diseases, State Key Laboratory of Cardiovascular Disease, Fuwai Hospital, National Center for Cardiovascular Diseases, Chinese Academy of Medical Sciences and Peking Union Medical College, Beijing, China

**Keywords:** ApoB, ApoB/ApoA1, apolipoproteins, arteriosclerosis, retinal arteriosclerosis

## Abstract

**Background:**

Retinal arteriosclerosis is a non-invasive indicator for systemic microvascular damage and early atherosclerosis. While traditional lipid measures are used to establish cardiovascular risk predictors, Apolipoprotein B (ApoB), Apolipoprotein A1 (ApoA1), and their ratio might provide more insights into the atherogenic burden. However, their association with retinal arteriosclerosis in general health populations has not yet been explored.

**Methods:**

This cross-sectional study included 4,938 adults from a health examination cohort. Retinal arteriosclerosis was evaluated by employing fundus photography and the SCHEIE classification. Multivariable logistic regression and restricted cubic spline analyses were used to evaluate the associations between apolipoproteins (ApoA1, ApoB/ApoA1 ratio) and retinal arteriosclerosis, accounting for demographic, lifestyle, clinical, and laboratory confounders.

**Results:**

In males, retinal arteriosclerosis prevalence was 13.98%, which is higher than females, who have 8.57%. People with retinal arteriosclerosis showed significantly higher ApoB levels and ApoB/ApoA1 ratios. In fully adjusted models, both ApoB (per 1-SD increase: OR 1.12, 95% CI 1.01–1.24) and ApoB/ApoA1 ratio (per 1-SD increase: OR 1.14, 95% CI 1.03–1.27) remained independently associated with retinal arteriosclerosis. Subgroup analyses revealed significant associations in adults ≥45 years, non-smokers, and non-hypertensive individuals.

**Conclusion:**

The ApoB/ApoA1 ratio is independently linked to retinal arteriosclerosis, especially in older adults. These findings indicate that integrating apolipoprotein profiling into cardiovascular risk assessment could improve the early detection of microvascular damage by leveraging the “eye-heart” axis.

## Introduction

Arteriosclerosis, as a systemic and chronic inflammatory disease of large and medium-sized arteries, usually progresses through a long subclinical phase before the clinical symptoms appear (1–4). Early identification of subclinical arteriosclerosis is therefore very important for effective cardiovascular risk stratification and prevention.

The retina is a unique part of the central nervous system that offers a non-invasive and direct view of the microvasculature. Retinal arterioles share similarities with cerebral and coronary arterioles in structure and function, like endothelial responsiveness and autoregulatory capacity. Therefore, retinal vasculature provides a unique, non-invasive view into systemic microcirculation, with clear links between retinal arteriolar changes and hypertension, metabolic disorders, and systemic arteriosclerosis (5–9).

Conventional lipid measurements, like low-density lipoprotein cholesterol (LDL-C) and high-density lipoprotein cholesterol (HDL-C), have served as a cornerstone in cardiovascular disease (CVD) risk assessment (10–12). But cholesterol concentration might not fully represent the actual number of atherogenic lipoprotein particles. In recent years, apolipoproteins have attracted a lot of research interest, considering them as potential ways to refine risk prediction. Apolipoprotein B (ApoB) is the main structural protein found in all atherogenic lipoproteins (13), and apolipoprotein A1 (ApoA1) is the major component of HDL particles (14). The ApoB/ApoA1 ratio, which integrates both atherogenic and atheroprotective pathways, is a robust predictor of atherosclerotic cardiovascular events, including myocardial infarction and stroke (15–21).

However, there are no researches exploring the association between ApoA1, ApoB, and ApoB/ApoA1 ratio and retinal arteriosclerosis in large health-examination populations. Specifically, there is a question whether this association is independent of traditional risk factors. Exploring these connections might improve early risk stratification and give a reason for targeted preventive actions.

Therefore, utilizing cross-sectional data from a health examination cohort, this study aimed to investigate the relationship between the apolipoproteins and retinal arteriosclerosis. We hypothesized that a higher ApoB and ApoB/ApoA1 level would be independently associated with a greater likelihood of retinal arteriosclerosis. Our findings are expected to contribute to a novel, integrated assessment strategy for the early identification of high-risk individuals via the “eye-heart” axis.

## Methods

### Study population

**Figure 1:**
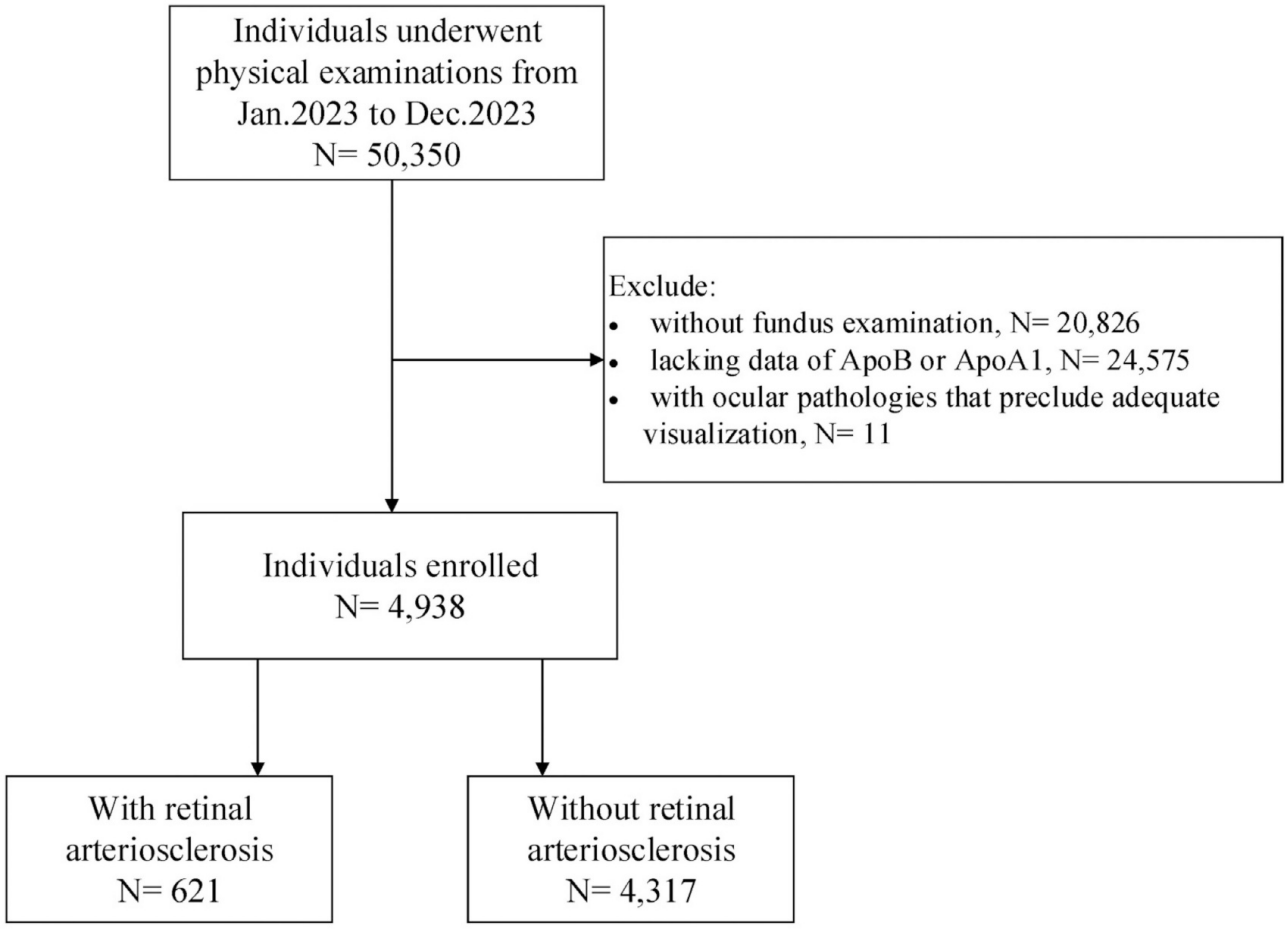
Flowchart of enrollment.

This study was carried out using data obtained from a health check-up network affiliated with the Healthcare Management Center of Peking University International Hospital, which offers annual comprehensive health evaluations to the community. The design and data sources have been reported in a previous publication (22). Briefly, the study included individuals aged 18 to 79 years who underwent examination between January 1st, 2023 and December 31st, 2023. From a total of 50,350 individuals, we excluded 20,826 individuals without retinal examination, 24,575 individuals lacking data on ApoB or ApoA1, and 11 individuals with ocular pathologies that preclude adequate visualization, such as cataract, vitreous hemorrhage, retinal detachment, post-panretinal photocoagulation, corneal leukoma, severe corneal edema, iridocyclitis, macular scarring, macular pucker affecting imaging, or significant vitreous opacities, resulting in 4,938 eligible participants (Figure 1).

The study was approved by the Ethics Committee of Peking University International Hospital (ethics number: 2023-KY-0045-01). Given that the analyses employed only de-identified data, the requirement for individual consent was waived by the Ethics Committee. All methods were performed in accordance with the Declaration of Helsinki and relevant guidelines. The personal information of the study subjects was confidential.

### Data collection and definition of risk factors

Data were extracted from electronic medical records. Demographic and clinical information was collected by trained staff following standardized protocols. Missing values were imputed using a regression approach that incorporated demographic data, smoking and alcohol consumption status, and medication history.

Height and weight were measured with participants standing erect without shoes and headwear. Body mass index (BMI) was calculated as weight in kilograms divided by height in meters squared (kg/m^2^). Hypertension was defined as systolic blood pressure ≥140 mmHg, diastolic blood pressure ≥90 mmHg, a prior physician diagnosis, or current use of antihypertensive medication. Diabetes mellitus was defined as fasting blood glucose ≥7.0 mmol/L, a prior physician diagnosis, or current use of hypoglycemic agents.

Venous blood samples were collected after a fasting period of at least 8 h. Serum concentrations of total bilirubin (TBIL), alanine aminotransferase (ALT), aspartate aminotransferase (AST), gamma-glutamyl transferase (GGT), albumin, globulin, total protein (TP), total cholesterol (TC), triglycerides (TG), LDL cholesterol (LDL-C), HDL cholesterol (HDL-C), ApoB, ApoA1, fasting plasma glucose (FPG), uric acid (UA), and blood urea nitrogen (BUN) were measured using an automated biochemistry analyzer. The ApoB/ApoA1 ratio was derived by dividing the ApoB value by the ApoA1 value.

### Measurement of retinal arteriosclerosis

The primary outcome was the presence of retinal arteriosclerosis. Retinal examination was performed by a professional ophthalmologist using a non-dilated fundus camera (AFC-330, NIDEK) according to a standard protocol (23). Grading was based on the SCHEIE classification (24):

Grade 0: no changes;

Grade 1: mild widening of the arterial light reflex and minimal arteriovenous (AV) crossing alterations;

Grade 2: bright, “copper wire” appearance of the arterioles with definite AV nicking (Salus's sign);

Grade 3: prominent “silver wire” appearance of the arterioles and definite attenuation of the venules at the AV crossings; and

Grade 4: all the changes of Grade 3, plus the presence of retinal hemorrhages, microaneurysms, and/or exudates indicative of ischemic retinal damage.

In this study, participants with features corresponding to Grade 1 through 4 were considered to have retinal arteriosclerosis.

### Statistical analyses

Continuous variables were presented as mean ± standard deviation for normally distributed data and as median (interquartile range) for skewed data. Categorical variables were expressed as numbers (percentages). Baseline characteristics between participants with and without retinal arteriosclerosis were compared using standardized mean difference (SMD) to assess the balance between groups, with an SMD > 0.2 indicating a meaningful difference.

The associations between apolipoproteins (including ApoA1, ApoB, and the ApoB/ApoA1 ratio) and retinal arteriosclerosis were analyzed via multivariable logistic regression. Each index was assessed as a continuous variable, categorized into three quartiles, and modeled as a non-linear function using a restricted cubic spline function with three internal knots and the median value serving as a reference point. A series of models were developed to adjust for potential confounders: Model 1, adjusted for age and sex; Model 2, further adjusted for lifestyle factors (smoking, alcohol consumption), medical history (hypertension, diabetes, dyslipidemia, chronic kidney disease, fatty liver disease, cardiovascular disease, and cancer), vital signs (systolic and diastolic blood pressure, pulse), body mass index, waist circumference, and estimated glomerular filtration rate; Model 3, additionally adjusted for laboratory markers (white blood cell count, hemoglobin, platelet count, eosinophil count, liver enzymes, total protein, albumin, alkaline phosphatase, *γ*-glutamyl transferase, total bilirubin, and fasting blood glucose). Results are presented as odds ratios (ORs) with corresponding 95% confidence intervals (CIs).

Stratified analyses were conducted using the fully adjusted model (Model 3) to investigate potential effect modifications by key demographic and clinical factors, including age, sex, smoking status, alcohol consumption, body mass index, hypertension, and dyslipidemia. Given the exploratory nature of several subgroup and stratified analyses, multiple comparisons were performed without formal adjustment for multiplicity.

The use of standardized mean difference (SMD) for baseline comparisons was preferred over traditional *p*-values, as SMD is less influenced by large sample sizes and directly quantifies the magnitude of between-group differences. For non-linear modeling, restricted cubic splines (RCS) with three knots (at the 10th, 50th, and 90th percentiles) were used to enable flexible estimation of dose-response relationships without assuming linearity. All statistical analyses were conducted with SAS (version 9.4). A two-sided *p* value less than 0.05 was considered statistically significant.

## Results

### Characteristics of subjects

The study included 4,938 participants, with retinal arteriosclerosis prevalence rates of 13.98% (511/3,655) in men and 8.57% (110/1,283) in women. As shown in Table 1, significant differences in baseline characteristics were observed between participants with and without retinal arteriosclerosis (SMD > 0.2). Specifically, the sclerosis group was significantly older (55.76 ± 9.28 years vs. 41.81 ± 9.49 years) and had a higher proportion of males (82.29% vs. 72.83%). This group also exhibited a more adverse cardiometabolic risk profile, including higher BMI, waist circumference, systolic and diastolic blood pressure, fasting plasma glucose levels, and significantly greater prevalence of hypertension, diabetes, and dyslipidemia.

**Table 1:** Characteristics of all participants with and without retinal arteriosclerosis.

Characteristics	Overall	Retinal arteriosclerosis	No retinal arteriosclerosis	SMD
	*N* = 4,938	*N* = 621	*N* = 4,317	
Age, years	43.57 ± 10.53	55.76 ± 9.28	41.81 ± 9.49	1.49*
Sex				
Male	3,655 (74.02)	511 (82.29)	3,144 (72.83)	0.23*
Female	1,283 (25.98)	110 (17.71)	1,173 (27.17)	−0.23*
Smoking history	1,263 (25.58)	235 (37.84)	1,028 (23.81)	0.31*
Alcohol consumption	1,335 (27.04)	241 (38.81)	1,094 (25.34)	0.29*
Disease History				
Hypertension	612 (12.39)	251 (40.42)	361 (8.36)	0.80*
Dyslipidemia	158 (3.2)	41 (6.6)	117 (2.71)	0.19
Diabetes	228 (4.62)	105 (16.91)	123 (2.85)	0.48*
Coronary heart disease	86 (1.74)	38 (6.12)	48 (1.11)	0.27*
Chronic kidney disease	21 (0.43)	3 (0.48)	18 (0.42)	0.01
Stroke	17 (0.34)	9 (1.45)	8 (0.19)	0.14
Cancer or malignant tumors	67 (1.36)	20 (3.22)	47 (1.09)	0.15
Physical examination				
SBP, mmHg	121.71 ± 15.05	131.72 ± 15.62	120.27 ± 14.4	0.76*
DBP, mmHg	75.17 ± 11.51	81.71 ± 11.09	74.23 ± 11.26	0.67*
Pulse rate, per min	72.89 ± 9.99	72.1 ± 10.46	73.01 ± 9.92	−0.09
BMI, kg/m^2^	24.98 ± 3.76	26.43 ± 3.51	24.77 ± 3.75	0.46*
Waist circumference, cm	86.68 ± 10.66	91.92 ± 9.4	85.92 ± 10.62	0.60*
Fatty liver	145 (2.94)	37 (5.96)	108 (2.5)	0.17
Lab test				
White blood cell count, 10^9^/L	5.72 ± 1.47	5.96 ± 1.49	5.69 ± 1.46	0.18
Red blood cell count, 10^9^/L	4.86 ± 0.46	4.85 ± 0.42	4.87 ± 0.47	−0.03
Hemoglobin, g/L	146.55 ± 14.52	147.99 ± 13.28	146.34 ± 14.68	0.12
Platelet count, 10^9^/L	249.76 ± 56.88	241.03 ± 60.44	251.02 ± 56.25	−0.17
Total neutrophil count, 10^9^/L	3.37 ± 1.13	3.57 ± 1.17	3.35 ± 1.12	0.19
Total lymphocyte count, 10^9^/L	1.85 ± 0.52	1.85 ± 0.52	1.85 ± 0.52	0.00
Total eosinophil count, 10^9^/L	0.11 (0.07, 0.18)	0.12 (0.07, 0.2)	0.1 (0.06, 0.17)	0.14
Total basophil count, 10^9^/L	0.02 (0.01, 0.04)	0.03 (0.01, 0.04)	0.02 (0.01, 0.04)	0.07
ALT,U/L	22 (16, 33)	23 (17, 32)	22 (15, 33)	−0.02
AST,U/L	21 (18, 26)	22 (18, 27)	21 (18, 25)	0.12
Total protein,g/L,	72.57 ± 4.12	71.78 ± 4.39	72.68 ± 4.06	−0.21*
Albumin,g/L	45.44 ± 2.44	44.76 ± 2.33	45.53 ± 2.44	−0.32*
Globulin,g/L	27.13 ± 3.49	27.01 ± 3.86	27.14 ± 3.44	−0.04
ALP, U/L	71.98 ± 19.02	76.11 ± 22.04	71.38 ± 18.48	0.23*
GGT,U/L	25 (17, 39)	29 (21, 44)	25 (17, 38)	0.15
Total bilirubin, umol/L	14 ± 5.76	14.56 ± 6.95	13.92 ± 5.56	0.10
Direct bilirubin,umol/L	4.7 ± 1.78	4.8 ± 1.82	4.68 ± 1.77	0.07
eGFR, mL/min/1.73m^2^	98.39 ± 16.2	94.67 ± 17.7	98.93 ± 15.9	−0.25*
eGFR＜60	20 (0.41)	6 (0.97)	14 (0.32)	0.08
FPG, mmol/L	5.33 ± 1.31	6.18 ± 1.95	5.21 ± 1.14	0.60*
TC, mmol/L	4.78 ± 0.92	4.8 ± 1.04	4.77 ± 0.9	0.03
TG, mmol/L	1.26 (0.83, 1.95)	1.48 (1.07, 2.22)	1.22 (0.81, 1.92)	0.21*
HDL-C, mmol/L	1.23 ± 0.3	1.17 ± 0.26	1.24 ± 0.3	−0.24*
LDL-C, mmol/L,	2.96 ± 0.79	2.96 ± 0.88	2.96 ± 0.78	0.00
LDL/HDL	2.54 ± 0.87	2.62 ± 0.88	2.53 ± 0.87	0.11
ApoA1, mg/dL	138.74 ± 22.05	138.95 ± 21.47	138.71 ± 22.13	0.01
ApoB, mg/dL	91.81 ± 23.88	96.55 ± 24.65	91.13 ± 23.69	0.22*
ApoB/ApoA1	0.68 ± 0.21	0.71 ± 0.2	0.67 ± 0.21	0.16

SMD, standardized mean difference; ALT, alanine aminotransferase; AST, aspartate aminotransferase; ALP, alkaline phosphatase; GGT, gamma-glutamyl transferase; TG, triglycerides; TC, total cholesterol; HDL-C, high-density lipoprotein cholesterol; LDL-C, low-density lipoprotein cholesterol; eGFR, estimated glomerular filtration rate; FPG, fasting plasma glucose; ApoB, apolipoprotein B; ApoA1, apolipoprotein A1; SBP, systolic blood pressure; DBP, diastolic blood pressure.

*absolute SMD > 0.2.

Regarding the core lipid parameters, the retinal arteriosclerosis group had significantly higher levels of ApoB compared to the control group (96.55 ± 24.65 vs. 91.13 ± 23.69 mg/dL) and a slightly higher ApoB/ApoA1 ratio (0.71 ± 0.20 vs. 0.67 ± 0.21). In contrast, the level of ApoA1 did not differ significantly between the two groups (138.95 ± 21.47 vs. 138.71 ± 22.13 mg/dL).

### Association of apolipoproteins and retinal arteriosclerosis

**Figure 2:**
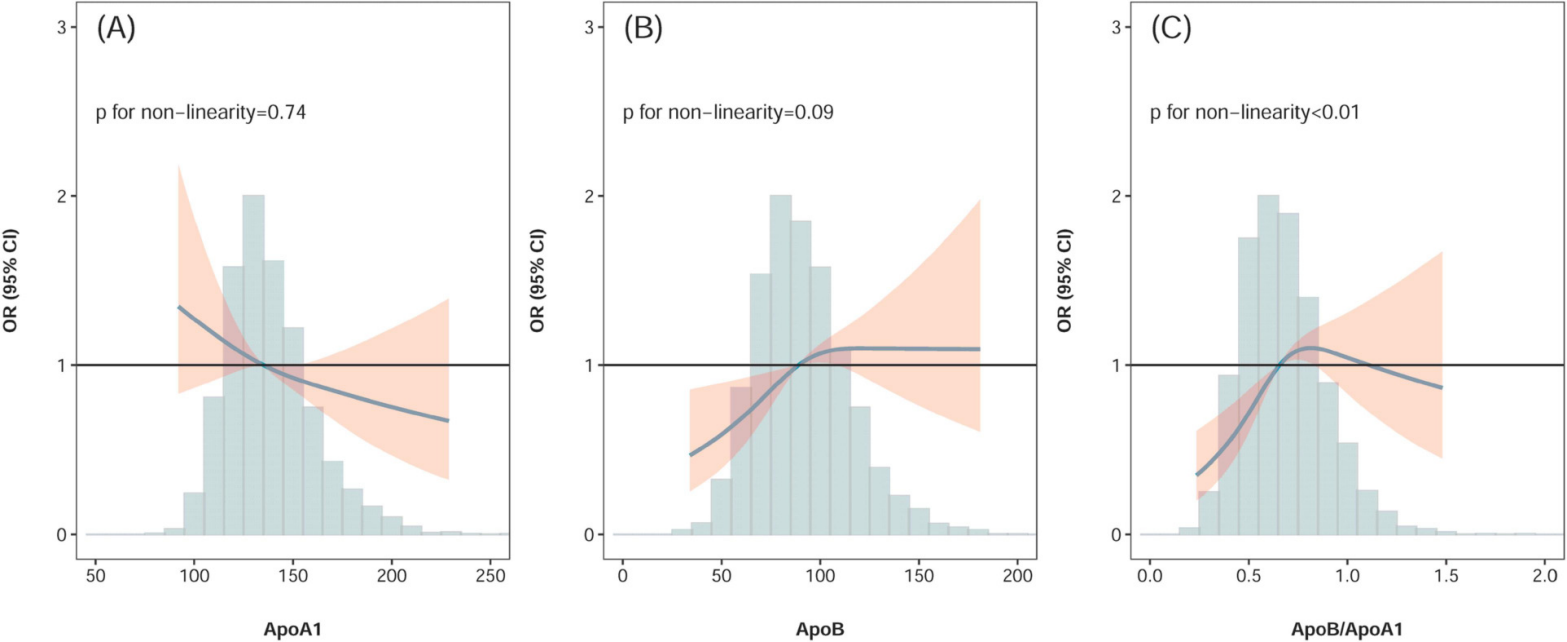
Restricted cubic splines of apolipoproteins and retinal arteriosclerosis. **(A)** ApoA1 **(B)** ApoB **(C)** ApoB/ApoA1. RCS model was built based on logistic model adjusting age, sex, lifestyle factors, medical history, vital signs, body mass index, waist circumference, estimated glomerular filtration rate and laboratory markers.

The results of the multivariable logistic regression analyses are detailed in Table 2. In the adjusted model 1, both ApoB and the ApoB/ApoA1 ratio were significantly associated with an increased risk of retinal arteriosclerosis, while ApoA1 had converse association. These associations in ApoB and the ApoB/ApoA1 ratio remained statistically significant after sequential adjustment for lifestyle factors, medical history, physical measurements, renal function, and a range of laboratory markers (Models 2 and 3). In the fully adjusted Model 3, both ApoB (per 1-SD increase: OR 1.12, 95% CI 1.01–1.24) and the ApoB/ApoA1 ratio (per 1-SD increase: OR 1.14, 95% CI 1.03–1.27) remained independently associated with the outcome. Analysis by tertiles revealed that participants in the highest tertile of ApoB had a 44% significantly increased risk of retinal arteriosclerosis compared to those in the lowest tertile (OR 1.44, 95% CI 1.1–1.89), while participants in the lowest tertile of ApoB/ApoA1 had a 36% lower risk compared to those in the middle tertile (OR 0.64, 95% CI 0.49–0.84), RCS analysis (Figure 2) further explored the possible nonlinear nature of these associations. ApoA1 showed a linear inverse association with the risk of retinal arteriosclerosis. ApoB exhibited a nonlinear positive association with risk, but this was not statistically significant. Notably, the ApoB/ApoA1 ratio demonstrated a significant converse U-shaped relationship with risk, where the likelihood of retinal arteriosclerosis reached the highest once the ratio exceeded approximately 0.75.

**Table 2:** Association of apolipoproteins and retinal arteriosclerosis.

	Adjusted model 1		Adjusted model 2		Adjusted model 3	
Variables	OR(95% CI)	*p* value	OR(95% CI)	*p* value	OR(95% CI)	*p* value
ApoA1						
per 10 mg/dL	0.93 (0.89,0.98)*	<0.01*	0.95 (0.91,1)	0.07	0.95 (0.9,1)	0.06
per 1SD	0.85 (0.77,0.95)*	<0.01*	0.9 (0.81,1.01)	0.07	0.9 (0.8,1.01)	0.06
Q1	Ref		Ref		Ref	
Q2	0.85 (0.67,1.08)	0.19	0.86 (0.66,1.11)	0.23	0.83 (0.64,1.07)	0.15
Q3	0.8 (0.63,1.03)	0.08	0.85 (0.66,1.12)	0.25	0.83 (0.64,1.09)	0.19
ApoB						
per 10 mg/dL	1.07 (1.03,1.11)*	<0.01*	1.05 (1.01,1.1)*	0.02*	1.05 (1,1.09)*	0.04*
per 1SD	1.17 (1.06,1.28)*	<0.01*	1.13 (1.02,1.25)*	0.02*	1.12 (1.01,1.24)*	0.04*
Q1	Ref		Ref		Ref	
Q2	1.13 (0.88,1.46)	0.35	1.13 (0.86,1.48)	0.39	1.13 (0.86,1.5)	0.37
Q3	1.53 (1.2,1.95)*	<0.01*	1.45 (1.12,1.88)*	<0.01*	1.44 (1.1,1.89)*	<0.01*
ApoB/ApoA1						
per 0.1	1.09 (1.05,1.15)*	<0.01*	1.07 (1.02,1.12)*	<0.01*	1.07 (1.01,1.12)*	0.01*
per 1SD	1.21 (1.1,1.33)*	<0.01*	1.15 (1.04,1.28)*	<0.01*	1.14 (1.03,1.27)*	0.01*
Q1	0.65 (0.51,0.84)*	<0.01*	0.65 (0.5,0.85)*	<0.01*	0.64 (0.49,0.84)*	<0.01*
Q2	Ref		Ref		Ref	
Q3	1.13 (0.9,1.42)	0.28	1.03 (0.81,1.31)	0.8	1.01(0.79,1.29)	0.92

Model 1, adjusted for age and sex; Model 2, further adjusted for lifestyle factors, medical history, vital signs, body mass index, waist circumference, and estimated glomerular filtration rate; Model 3, additionally adjusted for laboratory markers.

*p value < 0.05.

### Stratified analyses

**Figure 3:**
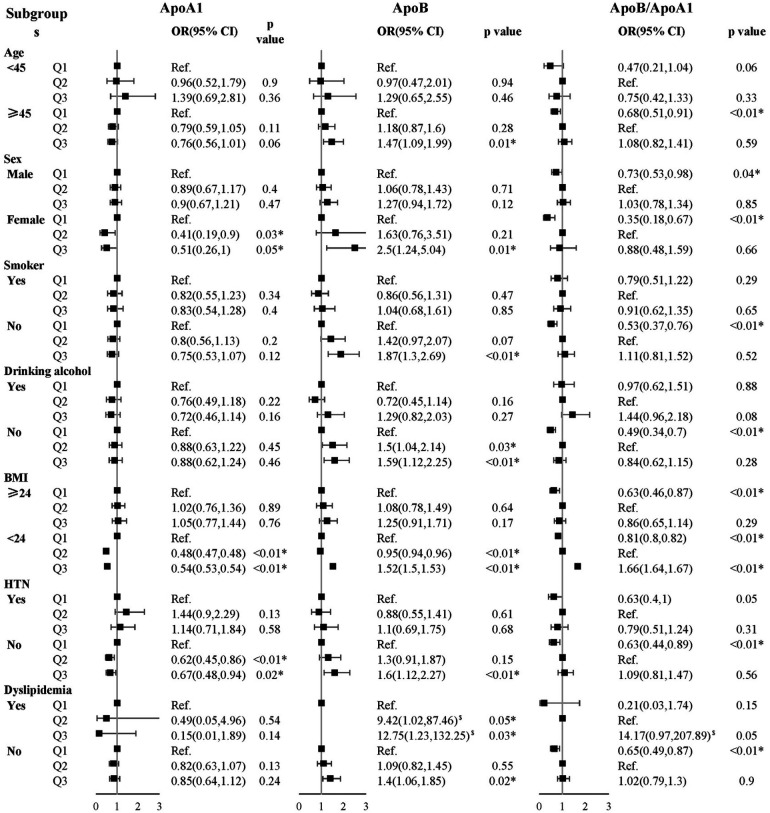
Association of apolipoproteins and retinal arteriosclerosis in subgroups. * *p* value < 0.05. ^$^ The odds ratio is not displayed for out of range of the axes. Models were built based on logistic model adjusting age, sex, lifestyle factors, medical history, vital signs, body mass index, waist circumference, estimated glomerular filtration rate and laboratory markers.

Stratified analyses using the fully adjusted model demonstrated potential for effect modification by key demographic and clinical factors (as shown in Figure 3), resulting in relatively stable outcomes across different groups.The significant positive associations of both ApoB and the ApoB/ApoA1 ratio with retinal arteriosclerosis were consistently observed among individuals aged ≥45 years, but were not observed in participants younger than 45 years. Moreover, smoking and drinking status changed the associations, since significance was limited to non-smokers and non-drinkers. In specific subgroups like individuals with BMI<24 kg/m2, females, and non-hypertensive participants, higher levels of ApoA1 were linked to a significant protective effect against retinal arteriosclerosis.

## Discussion

In a large cross-sectional study of a health examination population, we examined the specific relationships between apolipoproteins (ApoB, ApoA1, and their ratio) and retinal arteriosclerosis. Our main findings are three. First, baseline comparisons showed that people with retinal arteriosclerosis had a much more adverse cardiometabolic profile, which includes higher levels of ApoB and a higher ApoB/ApoA1 ratio, but not ApoA1. Second, in multivariable logistic regression analyses, both ApoB and the ApoB/ApoA1 ratio showed significant, independent associations with retinal arteriosclerosis, even after extensive adjustment for a wide range of non-lipid confounders. Third, these associations were profoundly modified by key demographic and clinical characteristics, being particularly stable and consistent in older adults (≥45 years), females, non-smokers, and individuals without hypertension.

The ApoB/ApoA1 ratio has a significant association with retinal arteriosclerosis, which is maintained after accounting for a lot of confounders in our fully adjusted model, suggesting it could be a better integrative marker for atherogenic lipid burden. ApoB is the total number of atherogenic lipoprotein particles (25), and ApoA1 is the main protein moiety in atheroprotective HDL particles (26). Therefore, the ratio represents the equilibrium between pro-atherogenic and anti-atherogenic forces. Our observation that the highest tertile of the ApoB/ApoA1 ratio is linked to a 36% reduction in the odds of sclerosis is generally consistent with this concept. This association is in line with a large body of evidence showing that the ApoB/ApoA1 ratio is a strong predictor of macrovascular events such as myocardial infarction (19), stroke (16), and major adverse cardiovascular events after percutaneous coronary intervention (17, 20). Our study extends its relevance to microvascular disease as manifested in the retina.

An intriguing finding in our study is that while LDL-C levels were nearly identical between participants with and without retinal arteriosclerosis (2.96 ± 0.88 vs. 2.96 ± 0.78 mmol/L, SMD = 0.00), significant differences were observed in ApoB (96.55 ± 24.65 vs. 91.13 ± 23.69 mg/dL), HDL-C (1.17 ± 0.26 vs. 1.24 ± 0.30 mmol/L), and the ApoB/ApoA1 ratio (0.71 ± 0.20 vs. 0.67 ± 0.21). This apparent discordance highlights a well-recognized limitation of LDL-C as a stand-alone marker. LDL-C measures cholesterol content per particle but does not reflect the total number of atherogenic lipoprotein particles (27, 28). In contrast, ApoB directly quantifies the particle number of all atherogenic lipoproteins [very low-density lipoprotein, intermediate-density lipoprotein, LDL and lipoprotein(a)] (29). Individuals can have similar LDL-C levels but markedly different ApoB concentrations due to variations in particle size and composition – a phenomenon often referred to as ‘discordance’ (30)This arises mainly from variations in triglyceride metabolism, cholesteryl ester transfer protein (CETP)-mediated lipid exchange, and insulin resistance, which alter LDL particle size and cholesterol content per particle (13, 30–33). The presence of retinal arteriosclerosis was associated with higher ApoB (i.e., more atherogenic particles) despite similar LDL-C, suggesting that particle number may be a more sensitive indicator of subclinical microvascular damage than cholesterol concentration. Furthermore, lower HDL-C in the sclerosis group reflects reduced atheroprotective capacity. Thus, our findings reinforce the concept that apolipoprotein profiling (ApoB and ApoB/ApoA1 ratio) provides complementary information beyond traditional lipid panels, particularly in populations where LDL-C appears unremarkable but residual microvascular risk persists.

Retinal vasculature is a unique, directly observable window into the body's microcirculation, which has embryological, anatomical, and physiological similarities to cerebral and coronary arteries. This foundational concept is strongly supported by prior research, which has shown that retinal microvascular abnormalities are associated with angiographically proven coronary artery disease (5), predict long-term cardiovascular outcomes (6), correlate with coronary artery calcium scores (7), and are independently linked to carotid atherosclerosis (8) and the severity of coronary lesions (9).Longitudinal data from the Arteriosclerosis Risk in Communities (ARIC) Study showed that retinal vascular caliber measurements are significantly associated with the long-term risk of coronary heart disease (34). A landmark study by Rim et al. demonstrated that a deep-learning model analyzing retinal photographs could directly predict coronary artery calcium scores, which is a validated measure of coronary atherosclerotic burden. Although CAC (coronary artery calcium) score is a marker of calcified plaque, the study showed that retinal-based risk stratification performed comparably to CT-measured CAC in predicting future cardiovascular events, indicating the retina plays a role as a window to systemic vascular health (7). A large cross-sectional study by Liu et al. involving over 20,000 Chinese individuals showed that fundus arteriosclerosis was independently associated with an increased risk of carotid arteriosclerosis (8). Furthermore, cutting-edge studies utilizing swept-source optical coherence tomography angiography (SS-OCTA) have quantitatively confirmed that impairments in the retinal microcirculation, like reduced vessel density, are closely linked to the presence and severity of coronary artery disease (9). Our findings provide a key biochemical link to this anatomical and functional evidence. We show that an imbalance in circulating atherogenic lipids, quantified by the ApoB/ApoA1 ratio, is independently associated with structural retinal arteriolar changes (arteriosclerosis). This relationship between a blood-based lipid marker and a direct microvascular assessment supports the retina as a reliable indicator of systemic vascular health, and provides a more comprehensive, non-invasive method for early risk identification using the “eye-heart” axis.

The significant association between dyslipidemia and retinal arteriosclerosis in individuals ≥45 years, but not in those <45 years, likely reflects low statistical power, resulting from the low baseline prevalence of retinal arteriosclerosis in younger group. The significant associations are confined to non-smokers and non-hypertensive individuals, which is intriguing. The observed pattern can be attributed to the influence of competing risk factors. In smokers (35), the direct vascular damage caused by smoking might reduce the relative contribution of ApoB/ApoA1, thereby obscuring its statistical association. Conversely, in the low-risk group without smoking, ApoB/ApoA1 is a dominant and unmasked pathogenic driver, so its association is statistically significant. As for drinking alcohol, its relation to retinal arteriosclerosis is complicated, because alcohol has opposing effects on different risk factors (36–38). This duality creates conflicting biological influences, making the net clinical impact hard to define.

From a clinical standpoint, our data, combined with the known link between retinal changes and systemic arteriosclerosis, suggests that integrating routine fundus examination into the cardiovascular risk assessment workflow is a viable approach for high-risk people. It also considers apolipoprotein profiling, especially the ApoB/ApoA1 ratio, as a promising biomarker for detecting early microvascular damage, especially in populations where this association is most pronounced, like older individuals. This bidirectional approach is aligned with the increasing focus on personalized prevention, which could be integrated into existing risk prediction algorithms like the Pooled Cohort Equations or SCORE2 (12) and the China-PAR (Prediction for ASCVD Risk in China) model – which was developed and validated for Chinese adults (39). Additionally, the cost-effectiveness of a combined strategy needs evaluation, especially in populations with intermediate cardiovascular risk, where traditional markers might be ambiguous.

### Limitations

Our study has a few limitations. First, this study relies on the qualitative SCHEIE classification without quantitative vessel diameter measurement. This approach is subjective and prone to observer bias, and *non-differential misclassification would likely attenuate true associations, potentially underestimating the strength of the relationship between apolipoproteins and retinal arteriosclerosis.*The absence of standardized quantitative metrics, like arteriolar-to-venular ratios, also limits comparability with contemporary digital image analysis and integration into more advanced predictive models (6). *Therefore, our findings should be interpreted with caution, and future studies should employ quantitative vessel caliber measurement to reduce observer-dependent error.* However, this the most commonly used and cheapest examination method currently, so it's a feasible way to screen individuals at risk of arteriosclerosis. Second, its cross-sectional design prevents any causal inference about the temporal relationship between apolipoproteins and retinal arteriosclerosis. Even though there were comprehensive adjustments, the residual confounding factors like diet, physical activity, and genetic predisposition can't be excluded. Third, the possibility of chance findings due to multiple comparison cannot be excluded. The reported *p*-values should be interpreted with caution, and the results should be considered hypothesis-generating rather than confirmatory. Finally, the study population included health examination participants, which might restrict the generalizability to clinical or high-risk symptomatic groups.

## Conclusion

To summarize, this study demonstrated that the ApoB/ApoA1 ratio is an independent factor linked to retinal arteriosclerosis, a key indicator of systemic microvascular aging and damage, especially in older adult populations. These findings indicate that adding apolipoprotein profiling into cardiovascular risk assessment might improve early detection of individuals at high risk for microvascular damage, allowing for more targeted preventive measures (40). Future studies are needed to confirm these associations, to see if apolipoprotein-guided interventions can slow the progression of microvascular disease, and further delay the progression of systemic arteriosclerosis, thereby reducing the occurrence of major adverse cardiovascular and cerebrovascular events. Moreover, combining AI-based retinal image analysis with traditional methods could automate and standardize the detection of arteriolar changes, thereby reducing the subjectivity involved in the process and allowing for large-scale screening.

## Data Availability

The raw data supporting the conclusions of this article will be made available by the authors, without undue reservation.
